# Supplementary material for: Molecular and Morphological Characterization of Introgression Lines with Resistance to Bacterial Leaf Blight and Blast in Rice
Source: Plants (Basel). 2023 Aug 21;12(16):3012. doi: 10.3390/plants12163012 (PMC10458744; doi:10.3390/plants12163012)
Supplement: Supplementary file 1 [file plants-12-03012-s001.zip › plants-2386153-supplementary.pdf]

**Supplementary Table S1. Mean phenotypic performance of the yield traits in 46 rice genotypes during *kharif* 2020**

| S. No | Entry | DFF | PH | PL | TN | PN | GN | TW | PY |
|---|---|---|---|---|---|---|---|---|---|
| 1 | 19247 | 103 | 85 | 22.05 | 10 | 8 | 91 | 21.00 | 367 |
| 2 | 19013 | 89 | 86 | 22.20 | 10 | 12 | 81 | 25.25 | 363 |
| 3 | 19021 | 102 | 89 | 20.05 | 17 | 13 | 53 | 24.90 | 451 |
| 4 | 19022 | 101 | 89 | 19.40 | 21 | 21 | 92 | 25.65 | 581 |
| 5 | 19023 | 99 | 93 | 32.50 | 14 | 10 | 103 | 23.00 | 472 |
| 6 | 19024 | 99 | 88 | 21.75 | 16 | 14 | 83 | 22.75 | 504 |
| 7 | 19026 | 101 | 81 | 28.20 | 16 | 15 | 76 | 21.45 | 463 |
| 8 | 19180 | 110 | 98 | 24.50 | 10 | 10 | 93 | 27.95 | 430 |
| 9 | 19181 | 101 | 97 | 26.95 | 11 | 9 | 110 | 28.25 | 389 |
| 10 | 19182 | 109 | 85 | 25.65 | 19 | 16 | 118 | 24.60 | 444 |
| 11 | 19185 | 97 | 87 | 23.05 | 14 | 12 | 75 | 24.25 | 515 |
| 12 | 19206 | 98 | 92 | 21.60 | 11 | 12 | 55 | 24.80 | 399 |
| 13 | 19211 | 94 | 84 | 22.95 | 16 | 11 | 67 | 22.35 | 422 |
| 14 | 19396 | 97 | 84 | 22.15 | 16 | 12 | 92 | 23.95 | 608 |
| 15 | 19408 | 102 | 96 | 26.75 | 12 | 11 | 145 | 28.65 | 398 |
| 16 | 19411 | 98 | 79 | 22.85 | 16 | 11 | 99 | 23.00 | 447 |
| 17 | 19420 | 91 | 94 | 24.50 | 14 | 12 | 87 | 27.60 | 314 |
| 18 | 19461 | 101 | 95 | 27.65 | 13 | 10 | 133 | 29.45 | 341 |
| 19 | 19019 | 101 | 85 | 21.70 | 20 | 10 | 78 | 23.20 | 361 |
| 20 | 19020 | 102 | 89 | 20.30 | 26 | 20 | 67 | 24.45 | 476 |
| 21 | 19025 | 99 | 90 | 21.85 | 21 | 19 | 84 | 21.65 | 432 |
| 22 | 19030 | 101 | 93 | 21.50 | 10 | 9 | 159 | 21.45 | 756 |
| **23** | **RP1** | **98** | **84** | **21.15** | **16** | **15** | **105** | **24.70** | **4 73** |
| 24 | 19284 | 99 | 79 | 18.45 | 15 | 12 | 65 | 27.05 | 466 |
| 25 | 19345 | 119 | 88 | 32.00 | 10 | 8 | 163 | 14.90 | 308 |
| 26 | 19346 | 114 | 87 | 19.95 | 13 | 10 | 104 | 11.90 | 323 |
| 27 | 19484 | 101 | 98 | 22.85 | 10 | 9 | 96 | 16.15 | 426 |
| 28 | 19485 | 106 | 98 | 21.10 | 12 | 10 | 93 | 14.75 | 457 |
| 29 | 19128 | 103 | 79 | 24.00 | 11 | 7 | 241 | 20.95 | 299 |
| 30 | 19353 | 102 | 113 | 23.55 | 10 | 9 | 163 | 26.40 | 250 |
| 31 | 19072 | 108 | 104 | 25.30 | 13 | 11 | 161 | 12.15 | 542 |
| 32 | 19103 | 102 | 99 | 20.95 | 17 | 13 | 102 | 23.65 | 515 |
| 33 | 19144 | 106 | 87 | 18.80 | 14 | 12 | 152 | 16.20 | 414 |
| 34 | 19162 | 109 | 84 | 19.25 | 8 | 7 | 86 | 23.85 | 369 |
| 35 | 19283 | 101 | 79 | 16.45 | 13 | 13 | 111 | 23.15 | 358 |
| 36 | 19344 | 119 | 85 | 21.00 | 13 | 12 | 114 | 26.95 | 391 |
| 37 | 19347 | 119 | 93 | 20.20 | 10 | 6 | 132 | 15.90 | 440 |
| 38 | 19429 | 102 | 98 | 21.05 | 11 | 9 | 111 | 15.20 | 385 |
| 39 | 19447 | 97 | 89 | 21.65 | 11 | 9 | 86 | 19.65 | 382 |
| 40 | 19448 | 94 | 98 | 22.65 | 12 | 6 | 122 | 20.00 | 372 |
| 41 | 19483 | 102 | 109 | 21.40 | 11 | 10 | 175 | 17.15 | 520 |
| 42 | 19487 | 101 | 99 | 20.95 | 15 | 15 | 143 | 15.30 | 447 |
| **43** | **RP2** | **111** | **95** | **23.50** | **10** | **9** | **114** | **16.75** | **427** |
| 44 | C1 | 106 | 81 | 20.40 | 12 | 8 | 149 | 12.25 | 404 |
| 45 | C2 | 111 | 77 | 21.55 | 14 | 6 | 148 | 14.60 | 690 |
| 46 | C3 | 118 | 86 | 22.75 | 20 | 19 | 133 | 17.05 | 585 |
| | Mean | 103 | 90 | 23 | 12 | 12 | 111 | 21 | 439 |
| | Minimum | 89 | 77 | 16.45 | 6 | 6 | 53 | 11.90 | 250 |
| | Maximum | 119 | 113 | 32.50 | 21 | 26 | 241 | 29.45 | 756 |
| | Variance | 49.03 | 50.52 | 6.55 | 3.78 | 7.81 | 877.29 | 21.08 | 7373.76 |
| | SD | 7 | 7.11 | 2.56 | 1.94 | 2.79 | 29.62 | 4.59 | 85.87 |
| | SE | 1.03 | 1.05 | 0.38 | 0.29 | 0.41 | 4.37 | 0.68 | 12.66 |

| S. No | Entry | DFF | PH | PL | TN | PN | GN | TW | PY |
|---|---|---|---|---|---|---|---|---|---|
| | CV% | 6.79 | 7.91 | 11.35 | 15.63 | 22.65 | 26.63 | 21.43 | 19.57 |

DFF-Days to Fifty Percent Flowering, PH- Plant Height, PL- Panicle Length, TN-Tiller Number, PN-Panicle Number, GN- Grain Number, TW-Thousand grain weight, PY- Plot yield/ grain yield, SD- Standard Deviation, SE- Standard Error, CV% - Coefficient of Variation, RP1-Krishna Hamsa, RP2-WGL14, C1-Improved Samba Mashuri, C2-Samba Mahsuri, C3-Swarna.

**Supplementary Table S2. Mean phenotypic values of yield traits in 46 rice genotypes during *rabi* 2021**

| S.No | Entry | DFF | PH (cm) | PL (cm) | TN | PN | GN | TW (g) | PY (g/m$^2$) |
|---|---|---|---|---|---|---|---|---|---|
| 1 | 19247 | 129 | 80 | 21.86 | 13 | 12 | 91 | 19.56 | 805 |
| 2 | 19013 | 107 | 79 | 22.20 | 19 | 16 | 79 | 21.23 | 473 |
| 3 | 19021 | 117 | 76 | 21.19 | 17 | 17 | 97 | 20.41 | 803 |
| 4 | 19022 | 117 | 76 | 21.18 | 15 | 15 | 106 | 20.68 | 960 |
| 5 | 19023 | 112 | 78 | 21.80 | 17 | 17 | 120 | 21.20 | 896 |
| 6 | 19024 | 117 | 78 | 21.56 | 18 | 16 | 94 | 20.91 | 836 |
| 7 | 19026 | 101 | 74 | 21.25 | 19 | 17 | 90 | 20.91 | 671 |
| 8 | 19180 | 111 | 92 | 25.94 | 18 | 17 | 125 | 22.16 | 837 |
| 9 | 19181 | 111 | 89 | 26.48 | 17 | 16 | 113 | 25.23 | 797 |
| 10 | 19182 | 120 | 86 | 24.41 | 17 | 16 | 145 | 21.85 | 1018 |
| 11 | 19185 | 113 | 80 | 21.61 | 14 | 12 | 95 | 22.49 | 501 |
| 12 | 19206 | 108 | 76 | 22.04 | 17 | 14 | 86 | 19.83 | 417 |
| 13 | 19211 | 108 | 79 | 23.53 | 16 | 13 | 109 | 19.21 | 509 |
| 14 | 19396 | 112 | 79 | 20.68 | 18 | 18 | 66 | 21.20 | 687 |
| 15 | 19408 | 119 | 91 | 25.59 | 16 | 14 | 119 | 25.63 | 816 |
| 16 | 19411 | 114 | 82 | 22.50 | 16 | 15 | 93 | 21.16 | 663 |
| 17 | 19420 | 108 | 92 | 26.11 | 17 | 15 | 130 | 25.46 | 509 |
| 18 | 19461 | 128 | 96 | 24.75 | 17 | 15 | 125 | 19.70 | 734 |
| 19 | 19019 | 113 | 75 | 22.28 | 16 | 16 | 101 | 21.26 | 580 |
| 20 | 19020 | 116 | 79 | 22.35 | 19 | 18 | 116 | 20.65 | 780 |
| 21 | 19025 | 113 | 71 | 21.30 | 21 | 19 | 88 | 21.18 | 611 |
| 22 | 19030 | 110 | 89 | 21.28 | 18 | 17 | 136 | 18.90 | 744 |
| **23** | **RP1** | **105** | **77** | **21.39** | **16** | **14** | **96** | **20.03** | **474** |
| 24 | 19284 | 112 | 76 | 18.03 | 14 | 18 | 87 | 14.50 | 417 |
| 25 | 19345 | 128 | 90 | 22.43 | 14 | 13 | 145 | 13.15 | 471 |
| 26 | 19346 | 100 | 91 | 22.34 | 15 | 13 | 182 | 11.51 | 621 |
| 27 | 19484 | 128 | 88 | 21.78 | 19 | 14 | 159 | 14.88 | 905 |
| 28 | 19485 | 128 | 94 | 21.73 | 18 | 15 | 154 | 11.78 | 700 |
| 29 | 19128 | 128 | 85 | 20.74 | 14 | 14 | 83 | 19.63 | 720 |
| 30 | 19353 | 135 | 103 | 21.99 | 15 | 12 | 103 | 18.10 | 548 |
| 31 | 19072 | 112 | 97 | 27.18 | 14 | 14 | 177 | 12.71 | 410 |
| 32 | 19103 | 126 | 87 | 20.98 | 17 | 13 | 58 | 15.44 | 487 |
| 33 | 19144 | 126 | 98 | 20.78 | 18 | 16 | 128 | 18.56 | 619 |
| 34 | 19162 | 128 | 86 | 21.75 | 14 | 13 | 169 | 29.05 | 411 |
| 35 | 19283 | 105 | 73 | 18.98 | 18 | 15 | 90 | 12.85 | 434 |
| 36 | 19344 | 128 | 87 | 22.61 | 13 | 12 | 155 | 13.18 | 386 |
| 37 | 19347 | 134 | 92 | 23.05 | 14 | 13 | 114 | 12.54 | 271 |
| 38 | 19429 | 135 | 96 | 20.79 | 16 | 16 | 103 | 14.93 | 441 |
| 39 | 19447 | 103 | 90 | 20.24 | 17 | 12 | 92 | 18.86 | 323 |
| 40 | 19448 | 126 | 86 | 20.75 | 26 | 19 | 74 | 18.59 | 541 |
| 41 | 19483 | 100 | 97 | 20.08 | 16 | 14 | 97 | 14.64 | 710 |
| 42 | 19487 | 110 | 93 | 20.26 | 18 | 17 | 133 | 14.03 | 704 |
| **43** | **RP2** | **129** | **92** | **23.66** | **13** | **12** | **149** | **12.23** | **490** |
| 44 | C1 | 111 | 72 | 19.65 | 21 | 14 | 107 | 13.96 | 436 |
| 45 | C2 | 115 | 79 | 20.39 | 18 | 16 | 137 | 14.36 | 566 |
| 46 | C3 | 133 | 87 | 23.19 | 18 | 16 | 129 | 16.71 | 721 |

| S.No | Entry | DFF | PH (cm) | PL (cm) | TN | PN | GN | TW (g) | PY (g/m²) |
|---|---|---|---|---|---|---|---|---|---|
| | Mean | 117 | 85 | 22 | 16 | 16 | 114 | 18 | 609 |
| | Minimum | 100 | 71 | 18.03 | 12 | 12 | 58 | 11.51 | 49 |
| | Maximum | 135 | 103 | 27.18 | 26 | 21 | 182 | 29.05 | 1081 |
| | Variance | 102.49 | 51.19 | 2.88 | 1.93 | 2.96 | 550.88 | 16.06 | 29380 |
| | SD | 10.12 | 7.15 | 1.7 | 1.39 | 1.72 | 23.47 | 4.01 | 171.41 |
| | SE | 1.49 | 1.05 | 0.25 | 0.21 | 0.25 | 3.46 | 0.59 | 25.27 |
| | CV% | 8.64 | 8.4 | 7.65 | 8.77 | 10.93 | 20.67 | 21.74 | 28.17 |

DFF-Days to Fifty Percent Flowering, PH- Plant Height, PL- Panicle Length, TN-Tiller Number, PN-Panicle Number, GN- Grain Number, TW-Thousand grain weight, PY- Plot yield/ grain yield, SD- Standard Deviation, SE- Standard Error, CV% - Coefficient of Variation, RP1-Krishna Hamsa, RP2-WGL14, C1-Improved Samba Mashuri, C2-Samba Mahsuri, C3-Swarna.

**Supplementary Table S3. Pooled Mean phenotypic values of the yield traits in 46 rice genotypes**

| S. No | Entry | DFF | PH (cm) | PL (cm) | TN | PN | GN | TW (g) | PY (g/m²) |
|---|---|---|---|---|---|---|---|---|---|
| 1 | 19247 | 116 | 83 | 21.96 | 11 | 10 | 91 | 20.28 | 586 |
| 2 | 19013 | 98 | 82 | 22.20 | 15 | 14 | 80 | 23.24 | 418 |
| 3 | 19021 | 110 | 83 | 20.62 | 17 | 15 | 75 | 22.66 | 627 |
| 4 | 19022 | 109 | 83 | 20.29 | 18 | 18 | 99 | 23.16 | 771 |
| 5 | 19023 | 106 | 85 | 27.15 | 15 | 14 | 111 | 22.10 | 684 |
| 6 | 19024 | 108 | 83 | 21.66 | 16 | 16 | 88 | 21.83 | 670 |
| 7 | 19026 | 101 | 78 | 24.73 | 17 | 16 | 83 | 21.18 | 567 |
| 8 | 19180 | 111 | 95 | 25.22 | 14 | 13 | 109 | 25.06 | 633 |
| 9 | 19181 | 106 | 93 | 26.71 | 14 | 13 | 111 | 26.74 | 593 |
| 10 | 19182 | 115 | 85 | 25.03 | 17 | 17 | 131 | 23.23 | 731 |
| 11 | 19185 | 105 | 83 | 22.33 | 14 | 12 | 85 | 23.37 | 508 |
| 12 | 19206 | 103 | 84 | 21.82 | 14 | 13 | 70 | 22.32 | 408 |
| 13 | 19211 | 101 | 81 | 23.24 | 14 | 14 | 88 | 20.78 | 466 |
| 14 | 19396 | 105 | 81 | 21.41 | 17 | 15 | 79 | 22.58 | 647 |
| 15 | 19408 | 111 | 93 | 26.17 | 14 | 12 | 132 | 27.14 | 607 |
| 16 | 19411 | 106 | 81 | 22.68 | 15 | 13 | 96 | 22.08 | 555 |
| 17 | 19420 | 100 | 93 | 25.31 | 16 | 13 | 108 | 26.53 | 411 |
| 18 | 19461 | 115 | 95 | 26.20 | 15 | 12 | 129 | 24.58 | 537 |
| 19 | 19019 | 107 | 80 | 21.99 | 18 | 13 | 89 | 22.23 | 470 |
| 20 | 19020 | 109 | 84 | 21.33 | 23 | 19 | 92 | 22.55 | 628 |
| 21 | 19025 | 106 | 80 | 21.58 | 20 | 20 | 86 | 21.41 | 521 |
| 22 | 19030 | 106 | 91 | 21.39 | 14 | 13 | 147 | 20.18 | 750 |
| **23** | **RP1** | **102** | **80** | **21.27** | **15** | **5** | **101** | **22.36** | **474** |
| 24 | 19284 | 106 | 77 | 18.24 | 15 | 14 | 76 | 20.78 | 441 |
| 25 | 19345 | 124 | 89 | 27.21 | 11 | 11 | 154 | 14.03 | 390 |
| 26 | 19346 | 107 | 89 | 21.14 | 13 | 12 | 143 | 11.71 | 472 |
| 27 | 19484 | 115 | 93 | 22.31 | 14 | 12 | 127 | 15.51 | 666 |
| 28 | 19485 | 117 | 96 | 21.41 | 14 | 14 | 123 | 13.26 | 579 |
| 29 | 19128 | 116 | 82 | 22.37 | 12 | 10 | 162 | 20.29 | 509 |
| 30 | 19353 | 119 | 108 | 22.77 | 12 | 11 | 133 | 22.25 | 399 |
| 31 | 19072 | 110 | 100 | 26.24 | 13 | 12 | 169 | 12.43 | 476 |
| 32 | 19103 | 114 | 93 | 20.96 | 17 | 13 | 80 | 19.54 | 501 |
| 33 | 19144 | 116 | 93 | 19.79 | 15 | 15 | 140 | 17.38 | 516 |
| 34 | 19162 | 119 | 85 | 20.50 | 11 | 10 | 128 | 26.45 | 390 |
| 35 | 19283 | 103 | 76 | 17.71 | 16 | 14 | 101 | 18.00 | 396 |
| 36 | 19344 | 124 | 86 | 21.81 | 12 | 12 | 135 | 20.06 | 388 |
| 37 | 19347 | 127 | 92 | 21.63 | 11 | 10 | 123 | 14.22 | 355 |
| 38 | 19429 | 119 | 97 | 20.92 | 13 | 13 | 107 | 15.06 | 413 |
| 39 | 19447 | 100 | 90 | 20.94 | 13 | 11 | 89 | 19.26 | 353 |
| 40 | 19448 | 110 | 92 | 21.70 | 16 | 15 | 98 | 19.29 | 456 |

| S. No | Entry | DFF | PH (cm) | PL (cm) | TN | PN | GN | TW (g) | PY (g/m²) |
|---|---|---|---|---|---|---|---|---|---|
| 41 | 19483 | 101 | 103 | 20.74 | 13 | 12 | 136 | 15.89 | 615 |
| 42 | 19487 | 106 | 96 | 20.61 | 16 | 16 | 138 | 14.66 | 576 |
| **43** | **RP2** | **120** | **94** | **23.58** | **11** | **10** | **131** | **14.49** | **455** |
| 44 | C1 | 109 | 77 | 20.03 | 14 | 13 | 128 | 13.11 | 420 |
| 45 | C2 | 113 | 78 | 20.97 | 15 | 12 | 142 | 14.48 | 628 |
| 46 | C3 | 126 | 86 | 22.97 | 19 | 18 | 131 | 16.88 | 653 |
|  | Mean | 110 | 88 | 22 | 14 | 14 | 112 | 20 | 524 |
|  | Minimum | 98 | 76 | 17.71 | 11 | 10 | 70 | 11.71 | 238 |
|  | Maximum | 127 | 108 | 27.21 | 23 | 20 | 169 | 27.1 4 | 771 |
|  | Variance | 125 | 56 | 5 | 6 | 8 | 708 | 20.64 | 25446 |
|  | SD | 11.17 | 7.48 | 2.17 | 2.4 | 2.87 | 26.6 | 4.54 | 159.52 |
|  | SE | 1.16 | 0.78 | 0.23 | 0.25 | 0.3 | 2.77 | 0.47 | 16.63 |
|  | CV% | 10.14 | 8.55 | 9.69 | 17 | 20.45 | 23.67 | 22.8 | 30.46 |

DFF-Days to Fifty Percent Flowering, PH- Plant Height, PL- Panicle Length, TN-Tiller Number, PN-Panicle Number, GN- Grain Number, TW-Thousand grain weight, PY- Plot yield/ grain yield, SD- Standard Deviation, SE- Standard Error, CV% - Coefficient of Variation, RP1-Krishna Hamsa, RP2-WGL14, C1-Improved Samba Mashuri, C2-Samba Mahsuri, C3-Swarna.

**Supplementary Table S4. List of BB and BL gene specific primers used for molecular characterization**

| | | |
|---|---|---|
| *Xa21* | PTA248F | AGACGCGGAAGGGTGGTTCCCGGA |
| | PTA248R | AGACGCGGTAATCGAAAGATGAAA |
| *Xa33* | WRRM7.1F | TTTTATCCCCTTCTTCCTTC |
| | WRRM7.1R | CGTGTTTGTGTGTCTTTTG |
| | WRRM7.6F | CAACAAACACCTCCATGGTC |
| | WRRM7.6R | GGGAATGAGCAAAAATTGG |
| *xa13* | xa13ProF | GGCCATGGCTCAGTGTTTAT |
| | xa13ProR | GAGCTCCAGCTCTCCAAATG |
| *xa5* | xa5RF | AGCTCGCCATTCAAGTTCTTGAG |
| | xa5SRR | TGACTTGGTTCTCCAAGGCTT |
| | xa5SF | GTCTGGAATTTGCTCGCGTTCG |
| | xa5SR | TGGTAAAGTAGATACCTTATCAAACTGGA |
| *Xa38* | Oso4g53050-1F | TCTTCTATTGCTAACATTGGTG |
| | Oso4g53050-1R | TCGCATTCATTTTCAGAG |
| *xa23* | Lj74-F | AAGCCATTTGATGAGCAACC |
| | Lj74-R | GGATCCATTTCAGCATAACCTT |
| *Xa4* | RM224F | TCTCCCTCCTCCTCCTCCTACG |
| | RM224R | TGGTATAAAAGGCATTCGGG |
| *xa8* | RM21044F | GCAACTCGACGGAAGGCATCG |
| | RM21044R | GAGGGCTCAAGACGAACTCATCACG |
| | RM214F | CTGATGATAGAAACCTCTTCTC |
| | RM214R | AAGAACAGCTGACTTCACAA |
| *Xa27* | BDTG 19F | GAAGCCACACACACTGAGACA |
| | BDTG 19R | CGGAGGAGAACTAGAGAGACCA |
| | M1081F | TAGCTAAATAAAAGCAATTTTACGA |
| | M1081R | GCCCTTACATATCGATGTTTATTG |
| *Xa41* | pSWEET14F | ATTGGCACTTTCTGTCATGC |
| | pSWEET14R | GAGACCAAAGGCGAAGGCCCA |
| *Pi1* | RM224 F | CTCGATCGATCTTCACGAGG |
| | RM224 R | TGCTATAAAAGGCATTCGGG |
| *Pi2* | 195-1 F | ATGGTCCTTTATCTTTATTG |
| | 195-1 R | TTGCTCCATCTCCTCTGTT |
| *Pi9* | Nmsmpi9 F | CGAGAAGGACATCTGGTACG |
| | Nmsmpi9 R | GAGATGCTTGGTTTAGAAGAC |
| *Pi20* | RM1337 F | GCTGAGGAGTATCCTTTCTC |

| | | |
|---|---|---|
| | RM1337 R | ACCATAGGAAGATCATCACA |
| | RM5364 F | GTATTACGCTCGATAGCGGC |
| | RM5364 R | GTATCCTTTCTCGCAATCGC |
| *Pi38* | RM21F | ACAGTATTCCGTAGGCACGG |
| | RM21 R | GCTCCATGAGGGTGGTAGAG |
| *Pi54* | RM206 F | CCCATGCGTTTAACTATTCT |
| | RM206 R | CGTTCCATCGATCCGTATGG |
| | RM224 F | CTCGATCGATCTTCACGAGG |
| | RM224 R | TGCTATAAAAGGCATTCGGG |
| | PikhMAS F | CAATCTCCAAAGTTTTCAGG |
| | PikhMAS R | GCTTCAATCACTGCTAGACC |
| *Pib* | RM166 F | GGTCCTGGGTCAATAATTGGGTTACC |
| | RM166 R | TTGCTGCATGATCCTAAACCGG |
| | RM208 F | TCTGCAAGCCTTGTCTGATG |
| | RM208 R | TAAGTCGATCATTGTGTGGACC |
| *Pitp* | RM246 F | GAGCTCCATCAGCCATTCAG |
| | RM246 R | CTGAGTGCTGCTGCGACT |
| *Pizt* | Zt56591 F | TTGCTGAGCCATTGTTAAACA |
| | Zt56591 R | ATCTCTTCATATATATGAAGGCCAC |
| *Pi40* | pi40 F | CAACAAACGGGTCGACAAAGG |
| | pi40 R | CCCCCAGGTCGTGATACCTTC |
